# Influence of Neuropathology on Convection-Enhanced Delivery in the Rat Hippocampus

**DOI:** 10.1371/journal.pone.0080606

**Published:** 2013-11-08

**Authors:** Svetlana Kantorovich, Garrett W. Astary, Michael A. King, Thomas H. Mareci, Malisa Sarntinoranont, Paul R. Carney

**Affiliations:** 1 Department of Neuroscience, University of Florida, Gainesville, Florida, United States of America; 2 Wilder Center of Excellence for Epilepsy Research, University of Florida, Gainesville, Florida, United States of America; 3 Department of Pediatrics, Division of Pediatric Neurology, University of Florida, Gainesville, Florida, United States of America; 4 J. Crayton Pruitt Family Department of Biomedical Engineering, University of Florida, Gainesville, Florida, United States of America; 5 Department of Pharmacology and Therapeutics, University of Florida, Gainesville, Florida, United States of America; 6 Malcom Randall Veterans Affairs Medical Center, Gainesville, University of Florida, Gainesville, Florida, United States of America; 7 Department of Biochemistry and Molecular Biology, University of Florida, Gainesville, Florida, United States of America; 8 Department of Mechanical and Aerospace Engineering, University of Florida, Gainesville, Florida, United States of America; Universidade Federal do ABC, Brazil

## Abstract

Local drug delivery techniques, such as convention-enhanced delivery (CED), are promising novel strategies for delivering therapeutic agents otherwise limited by systemic toxicity and blood-brain-barrier restrictions. CED uses positive pressure to deliver infusate homogeneously into interstitial space, but its distribution is dependent upon appropriate tissue targeting and underlying neuroarchitecture. To investigate effects of local tissue pathology and associated edema on infusate distribution, CED was applied to the hippocampi of rats that underwent electrically-induced, self-sustaining status epilepticus (SE), a prolonged seizure. Infusion occurred 24 hours post-SE, using a macromolecular tracer, the magnetic resonance (MR) contrast agent gadolinium chelated with diethylene triamine penta-acetic acid and covalently attached to albumin (Gd-albumin). High-resolution T1- and T2-relaxation-weighted MR images were acquired at 11.1 Tesla in vivo prior to infusion to generate baseline contrast enhancement images and visualize morphological changes, respectively. T1-weighted imaging was repeated post-infusion to visualize final contrast-agent distribution profiles. Histological analysis was performed following imaging to characterize injury. Infusions of Gd-albumin into injured hippocampi resulted in larger distribution volumes that correlated with increased injury severity, as measured by hyperintense regions seen in T2-weighted images and corresponding histological assessments of neuronal degeneration, myelin degradation, astrocytosis, and microglial activation. Edematous regions included the CA3 hippocampal subfield, ventral subiculum, piriform and entorhinal cortex, amygdalar nuclei, middle and laterodorsal/lateroposterior thalamic nuclei. This study demonstrates MR-visualized injury processes are reflective of cellular alterations that influence local distribution volume, and provides a quantitative basis for the planning of local therapeutic delivery strategies in pathological brain regions.

## Introduction

With the growing development of nanoparticles, liposomes, proteins, and viral vectors as therapeutic agents for treating neurological disorders, novel bio-delivery strategies are necessary to target their transport to the central nervous system (CNS). Convection-enhanced delivery (CED), a local drug-delivery technique that uses positive pressure to deliver infusate directly into parenchymal interstitial space, is a promising technique for delivering therapeutic agents that do not readily cross the blood-brain-barrier (BBB). This method can yield high parenchymal drug concentrations and minimal systemic exposure without the limitations the BBB poses on size or chemical properties of therapeutic agents. For the biologics and therapeutic nanoparticles whose effective delivery to the CNS is problematic, CED is one of the only drug delivery strategies with the potential to provide uniform and large tissue distribution volumes, even compared with systemic delivery with injury breakdown of the BBB (for review, see 1–4). However, the efficacy of the procedure can be limited by poor targeting and the uncertain effects of tissue pathology on CED transport. Unexpected spread of therapeutics in the brain could lead to side effects and failure of clinical trials[5]. Since CED is used to deliver therapeutics into diseased and/or injured areas, it is necessary to fully understand how the delivery of various constructs may be affected under such conditions. We posit that a better understanding of brain tissue fluid dynamics will open a whole new class of drugs, devices, and systems for drug delivery in which brain access is currently off limits.

Previous studies in our group [6–8] and by others [9] have shown that infusate distributions in the hippocampus, a structure commonly affected in many neurological disorders, are influenced by specific characteristics of its underlying structure. We have comprehensively described both real-time [7,8] and final [6] infusate distributions within normal rat brains, establishing local tissue structure as a governing feature of CED distributions. These studies characterized interstitial flow in uninjured hippocampi, and results suggest pathological structural changes within the hippocampus would introduce variability in the distributions. It is clear that more detailed knowledge about how the anatomical and biophysical features of the brain affect infusate distribution is needed for optimization of delivery into diseased brain regions. CED has already been proposed as a novel therapeutic strategy to treat epilepsy [3], brain tumors [10], stroke [11], acute neuronopathic Gaucher disease [12], Parkinson's disease [13], Alzheimer's disease [14], and other localized neurodegenerative disorders; but no previous studies have investigated the effect of pathology on hippocampal distributions. Therefore, in an effort to optimize targeted delivery into injured regions, this study investigated the influence of microstructural changes in the hippocampus on the distribution of infusate using a model of acute brain injury. The self-sustaining status epilepticus (SE) experimental paradigm [15,16] was used in this study at 24 hours post-SE, when the molecular cascade involved in cerebral edema is underway. SE is a prolonged seizure that is well-documented to result in structural changes within the hippocampus [17–21] and represents a major risk factor for developing chronic temporal lobe epilepsy (TLE) [22–24]. Following SE brain injury, a cascade of ischemic and excitotoxic pathological events advance over time that are applicable to any disorder characterized by edema, BBB breakdown or inflammation. Therefore, this time point represents a manageable treatment window for prophylactic treatment of TLE; but it is also relevant for understanding effects of pathological changes occurring with general neurological injury and disease on drug delivery. 

In this study, SE brain injury was rated based on edema visualized with T2-weighted MR images, and characterized with staining against neuronal degeneration, myelin degradation, astrocytosis, and macrophage activation. CED infusions were performed 24 hours post-SE in the hippocampi of rodents; then MR imaging was used to monitor and measure the distribution of a non-binding protein tracer. This is an ideal model for macromolecular drug delivery and flow through the interstitial space due to low reactivity, convection-dominated transport, and ease of labeling with contrast agent. Infusate distributions in experimental animals from this study were compared to the results from control animal infusions completed previously [6].

Quantitative studies of distribution changes that occur with brain injury are sorely missing, but essential in any practical treatment planning application. The goal of this study is to provide foundational, quantitative data that adds to the current knowledge of factors influencing delivery of macromolecules within injured CNS regions. The predictive value of T2 information and underlying pathological influences on distribution spread measured in this study can be applied to improve targeting guidelines for CED, incorporated into injury-specific computational CED transport models developed by our group and others [7,25], and considered in delivery strategies of novel therapeutic agents, such as nanoparticles, viral vectors, anti-epileptogenic or neuroprotective substances [26–28], that could minimize or eliminate progressive structural injury.

## Methods

### Ethics Statement

All procedures were approved by the University of Florida Animal Care and Use Committee (Protocol # 201101784) and conducted in accordance with the National Institutes of Health Guide for the Care and Use of Experimental Animals. 

### Surgical Procedures

**Figure 1:**
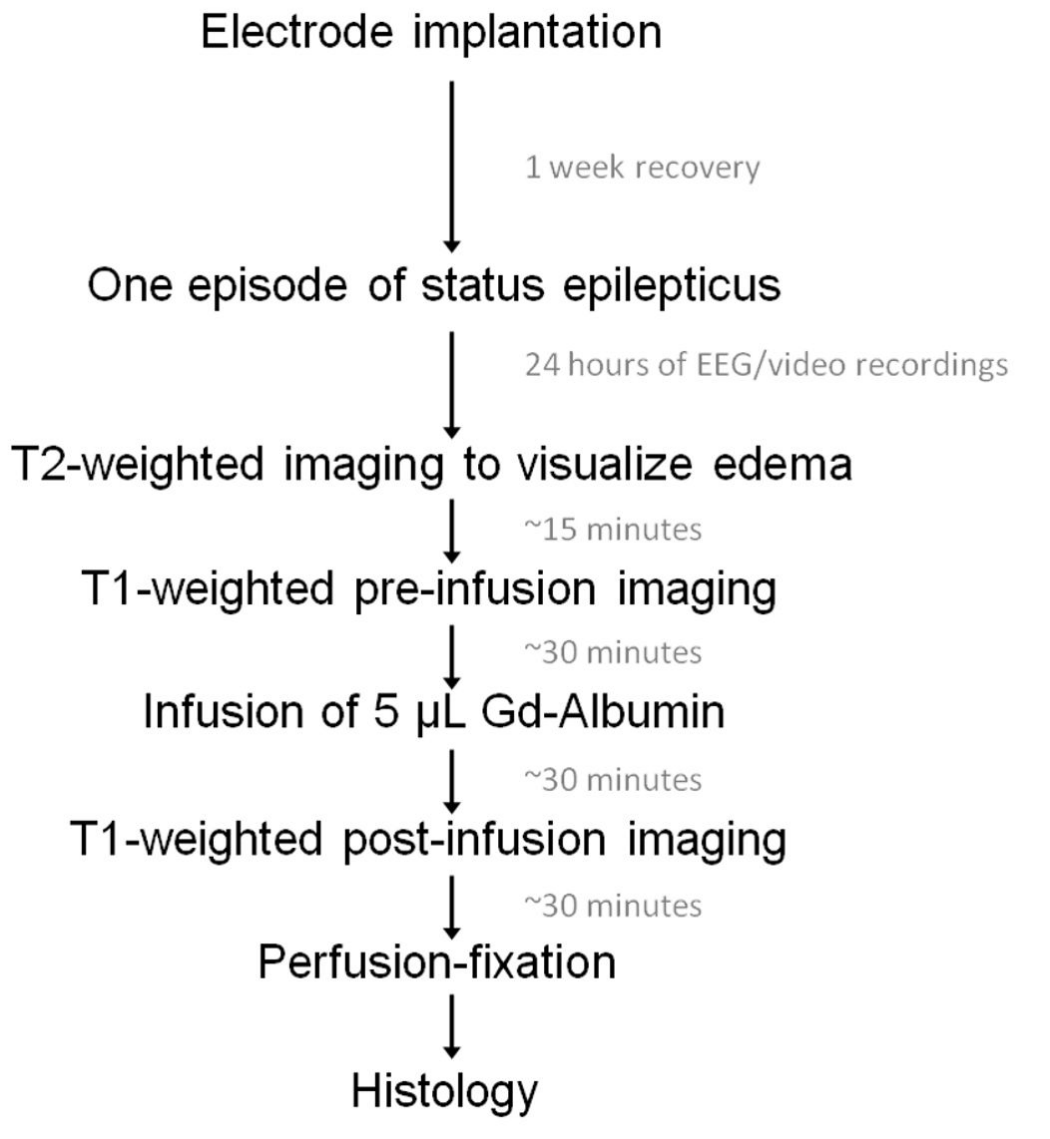
Experimental protocol flow chart.

A flow chart depicting the order of experiments can be seen in Figure 1. Male Sprague-Dawley rats (Harlan Labs, Indianapolis, IN) weighing 225-250 g on arrival were allowed one week to acclimate to the 12-h light/dark cycle and given food and water *ad libitum*. Anesthesia was initiated with xylazine (10 mg/kg, SQ) and 4% isoflurane in 1 L/min oxygen and maintained at 1.5% isoflurane in 0.4 L/min oxygen. Three 50 μm diameter polyamide-coated tungsten microwire electrodes (Plastics One, Roanoke VA) were implanted for the induction of self-sustaining SE as described in [17]. An electrode assembly of two electrodes was implanted into the right ventral hippocampus, flanking [-5.3AP, 4.9ML, 5.0DV] for stimulation and subsequent recording. A third electrode was implanted as a reference into the corpus callosum [-3.3AP, 4.9ML, 2.4DV]. A cannula guide was also secured to the skull region above the left dorsal hippocampus [-3.7AP, -2.2ML] for the future infusion of contrast agent. Four nylon anchoring screws were placed in the skull to allow for maximum support of the headset, which was permanently secured with Cranioplast cement (Plastics One, Roanoke VA). 

Previous studies by us [17,29–33] and others [34,35] have shown that electrode implantation alone does not result in structural damage or the development of spontaneous seizures. Additionally, infusion was planned contralateral to electrodes to avoid the physical confound of electrode implantation on infusate distribution. All animals were given at least one week to recover from the implantation surgery before the stimulation procedure began.

### Induction of structural brain injury by hippocampal electrical stimulation

One week post-electrode implantation, animals (n=26) were electrically stimulated to induce self-sustaining SE as described in [36]. Stimulus trains (50 Hz of 1ms biphasic square wave pulses) were delivered for 10s on and 2s off for a total of 78 ± 16 minutes. Animals were continuously recorded with time-locked video-EEG until 24 hours post-SE. A modified Racine scale [37,38] was used to grade the behavioral seizures as follows: grade 0 for no seizure response; grade 1 for immobility, eye closure, ear twitching, twitching of vibrissae, sniffing, facial clonus; grade 2 for head nodding associated with more severe facial clonus; grade 3 for clonus of one forelimb; grade 3.5 for bilateral forelimb clonus without rearing; grade 4 for bilateral forelimb clonus with rearing; grade 5 for rearing and losing balance. Animals (n=17) that experienced electrographic seizure activity for at least 2 hours post stimulation were included in the study to create a comparable injury across animals [17,31,34,36,39]. 

### MR Imaging

Twenty-four hours post induction of SE, high-resolution T1 and T2-weighted MR imaging was performed on animals (n=17) to generate a reference for contrast enhancement images (T1) and to visualize morphological changes (T2) pre-infusion. Immediately following CED of Gd-albumin (see next section), high-resolution T1-weighted imaging was repeated to visualize in vivo distribution profiles of the contrast agent in the rat brain. MR measurements were performed using a Bruker Avance imaging console (Bruker NMR Instruments, Billeria, MA) or Agilent Direct Drive imaging console (Agilent Technologies, Santa Clara, CA, USA) connected to a Magnex Scientific 11.1 T horizontal bore magnet system (Varian, Inc., Magnex Scientific Products, Walnut Creek, CA). A custom-made 130-degree arc, 3.5 cm rectangular linear-field surface coil constructed on a 4 cm diameter half-cylinder was used for linear transmission and detection of MR signal. High-resolution T1-weighted images, with slices oriented in the coronal direction, were acquired using a spin-echo sequence with a 2.5 cm×2.5 cm field-of-view in a matrix of 200×200, recovery time of 1000 ms, echo time of 10 ms, 8 averages and 30 slices, slice thickness 500 µm. T2-weighted data were acquired using a fast spin echo sequence with 30 slices, slice thickness 500 µm, oriented in the coronal direction and a 2.5 cm×2.5 cm field-of-view in a matrix of 208×208, recovery time of 3500 ms, RARE factor of 8 and effective echo time of 45 ms.

### T2 Measures of Edema and Injury Classification

T2-weighted images (30 coronal slices per brain) were examined and used to classify injury in animals (n=17). The high water content of edematous tissue results in prolonged T2 relaxation times and manifests as a hyperintense signal in T2-weighted images. Hyperintense signal was defined as injury in these images and then validated post-mortem (see *Immunohistochemistry*). These images were acquired prior to the introduction of the infusion cannula, thereby differentiating between SE-induced and cannula implantation injury. T2-weighted images were scored for injury as follows: Class 0 = control animals, no damage; Class 1 = unilateral piriform cortex and/or amygdala damage, Class 2 = score of 1 plus injury in the septal nuclei, Class 3 = a score of 2 plus injury in the middle thalamic nuclei, Class 4 = a score of 3 plus damage in the lateral thalamic nuclei, Class 5 = a score of 4 plus damage in the ventral subiculum, Class 6 = bilateral piriform cortex/amygdala damage, septal injury, and damage in the middle and lateral thalamic nuclei. An early indication of an injury severe enough to result in the development of spontaneous limbic seizures in this animal model is the presence of edema in the parahippocampal region [17]. To ensure a comparable injury across animals, those that did not exhibit parahippocampal edema were excluded from subsequent analyses.

### CED of Gd-albumin

Twenty-four hours post induction of SE, animals (n=17) were infused with 5.0 µL of the MR contrast agent diethylene triamine penta-acetic acid chelated gadolinium-labeled albumin (Gd-albumin, 10 mg/mL in PBS solution; MW ~87 kDa, ~35 Gd-DTPA molecules per albumin molecule; R. Brasch Laboratory, University of California, San Francisco, CA), tagged with Evans Blue dye (1 mg dye/50 mg Gd-albumin) into the dorsal dentate gyrus of the right hippocampus [-3.7 AP, -2.2 ML, -3.4 DV]. Evans Blue is a fluorescent dye that can be imaged with fluorescent microscopy to validate contrast agent distributions visualized with MR imaging [6]. The infusion was performed at 0.3 μL/min through the previously implanted cannula guide using a 100 μL gas-tight syringe (Hamilton, Reno, NV) driven by a syringe pump (Cole-Parmer, Vernon Hills, IL) connected to polyaryletheretherketone (PEEK) tubing (ID = 0.381 mm, OD = 0.794 mm, length ^~^ 0.5 m, Upchurch Scientific, Oak Harbor, WA). The PEEK tubing was coupled to a silica cannula (ID = 50 μm, OD = 147 μm, Polymicro Technologies, Phoenix, AZ) via a microfluidic connector.. Results from experimental animals infused in this study were compared to results from control animals infused under similar conditions in our previous study [6]. The only difference between infusions performed in control animals and experimental animals was an unavoidable additional ~30 minute time delay to transfer control animals to the MR imaging facility following infusion. 

### Immunohistochemistry

Following the last MR measurement, animals were transcardially perfused with 200 mL saline solution followed by 300 mL of 10% buffered formalin phosphate. Brains were extracted and stored in the formalin solution overnight at 4°C, then equilibrated in 30% sucrose solution for 72 hours. Brains were then sectioned coronally using a cryostat set at 50 µm. Every fourth section in succession was collected for staining to visualize degenerating neurons (Fluoro Jade C (FJC)), myelin (Black Gold II [40]), reactive astrocytes (glial fibrillary acidic protein (GFAP)), phagocytic microgliosis (CD-68). A modification by Lee et al [41] was used for FJC staining [42]. For assessment of micro- and astrogliosis, free-floating sections were incubated in 10mM citrate buffer, pH 9.0, for 25 minutes at 80°C for antigen retrieval. After a brief wash, they were incubated overnight in primary monoclonal antibodies against CD-68 (AbD Serotec; Raleigh, NC) or GFAP (G-A-5, Sigma Chemicals Co.; St. Louis, MO), at a concentration of 1:400. Sections were washed and incubated overnight in 1:10,000 biotinylated anti-mouse immunoglobulin G, reacted with a 1:1,000 Extravidin peroxidase solution for 2 hours, then visualized with 0.05% 3,3'-diaminobenzidine (DAB) in 0.0012% hydrogen peroxide in PBS. GFAP and CD-68-stained sections were counterstained with cresyl violet for visualization of cell bodies.

### Image Segmentation and Statistical Analysis

 Final distribution volumes of Gd-albumin were analyzed by performing semi-automatic image segmentation on the T1-weighted coronal images using routines written in MATLAB (The MathWorks Inc., Natick, MA, USA) with the following specific threshold criteria. Voxels were included in the infusion volume if their signal intensity was higher than at least 6 standard deviations of the noise in the corresponding region contralateral to the site of infusion (control regions containing no Gd-albumin). The segmentation output of the MATLAB routine was refined using the ITK-SNAP open-source medical image segmentation tool [43]. Dynamic and final distribution volumes in the dorsal and ventral hippocampus were calculated by counting the number of voxels included in each segmented region and multiplying by the volume of a single voxel. 

Total hippocampal volumes were calculated by manually segmenting the T2 weighted pre-infusion images in ITK-SNAP. The borders of the hippocampus (e.g. corpus callosum, thalamus) were determined by white matter/gray matter contrast in the T2 weighted images, anatomical landmarks such as the velum interpositum and by referring to a rat brain atlas [44].

 Infusate distribution volumes were compared to injury ratings using Kendall’s rank correlation [45]. This non-parametric test is used for ordinal data and analogous to Spearman’s rank correlation [46] for parametric data. The Kendall’s tau correlation coefficient was chosen over Spearman’s rho because tau is a better estimate of the corresponding population parameter and has more accurate p values in small sample sizes [47]. Furthermore, Spearman’s is difficult to interpret as a measure of the strength of a relationship and does not have a meaningful operational interpretation [48]. The significance of differences in volume of distributions between control animals and injury classifications of animals 24 hours post-SE was calculated by analysis of variance (ANOVA). Post hoc testing for individual classification differences was done with Newman-Keuls test [49]. All tests were two-tailed; a p<0.05 was considered significant.

## Results

### Brain injury

**Figure 2:**
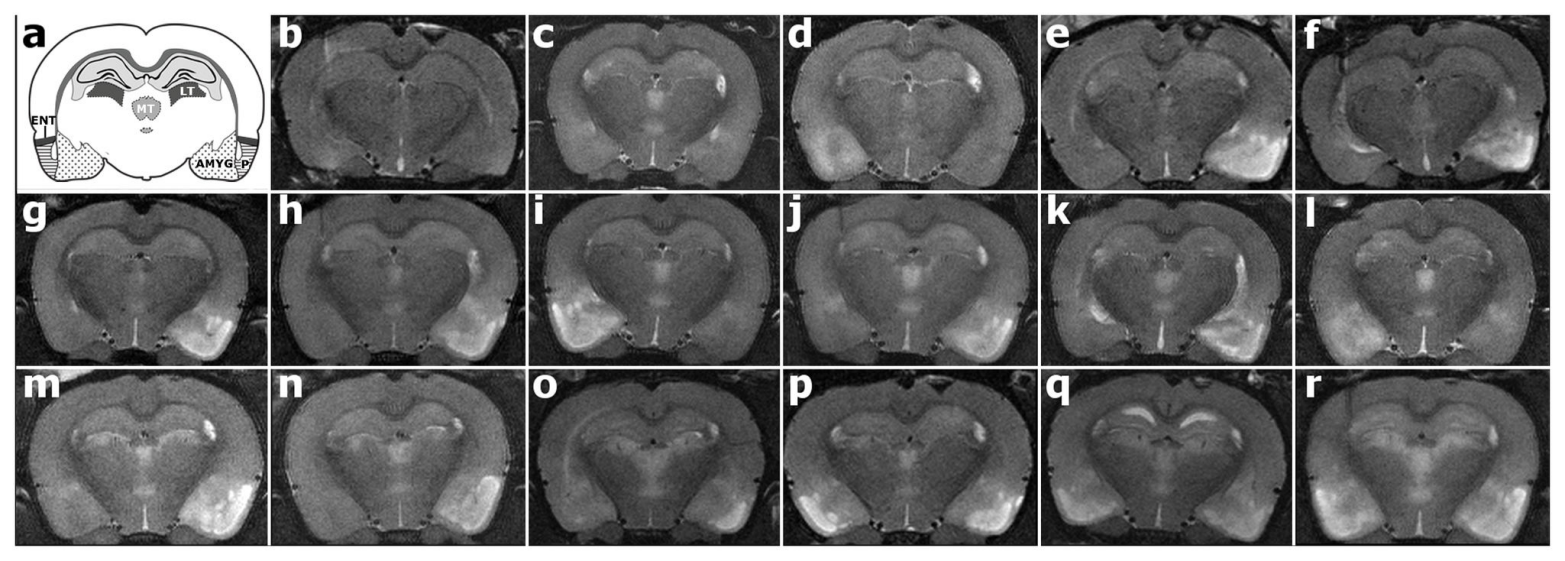
T2-weighted coronal images of 17 different rodent brains acquired 24 hours post induction of status epilepticus reveal injury within regions of the limbic circuitry. Hyperintense regions signify areas of injury. **A**: Schematic of affected structures adapted from [44]. Structures most affected were the CA3 hippocampal subfield, ventral subiculum, piriform cortex (P), entorhinal cortex (ENT), amygdalar nuclei (AMYG), middle thalamic nuclei (MT), and laterodorsal/lateroposterior thalamic nuclei (LT). **B**-**R**: MR images of injury induced post-SE. Electrode implantation is on the left side of each image.

This study investigated the effects of edema on CED at 24 hours following SE brain injury. Animals (n=17) were continuously electrically stimulated in the ventral hippocampus to experience one episode of SE. T2-weighted images (Figure 2) were acquired 24 hours post induction of SE to reveal edema within regions of the limbic circuitry. One animal did not exhibit edema in the parahippocampal region and was not included in the subsequent injury analyses. Three of 16 animals exhibited edema in the bilateral parahippocampal region (Figure 2P-R) and 13 of 16 animals exhibited unilateral edema (Figure 2C-O). Of the animals that exhibited unilateral edema, injury was ipsilateral to the stimulating electrode in 3 of 13 animals (Figure 2D,I,L), and contralateral in 10 of 13 animals (Figure 2C,E-H,J-K,N-O). Table 1 presents the T2 injury index classification of each animal. 

**Table 1:** Index for injury classification 24 hours post status epilepticus.

**Injury Class**	**Injury Description**	**Number and % of animals per Class**
1	Unilateral edema in the piriform cortex and amygdalar nuclei	2 (12.5%)
2	Class 1 plus edema in septal nuclei	1 (6.25%)
3	Class 2 plus edema in the middle thalamic nuclei	2 (12.5%)
4	Class 3 plus edema in the laterodorsal/lateroposterior thalamic nuclei	5 (31.25%)
5	Class 4 plus edema in the ventral subiculum	3 (18.75%)
6	Class 5 plus bilateral edema in the piriform cortex and amygdalar nuclei	3 (18.75%)

Injured regions were identified using T2-weighted coronal images obtained in vivo prior to infusions.

### CED distribution volume and changes in hippocampal volume

**Figure 3:**
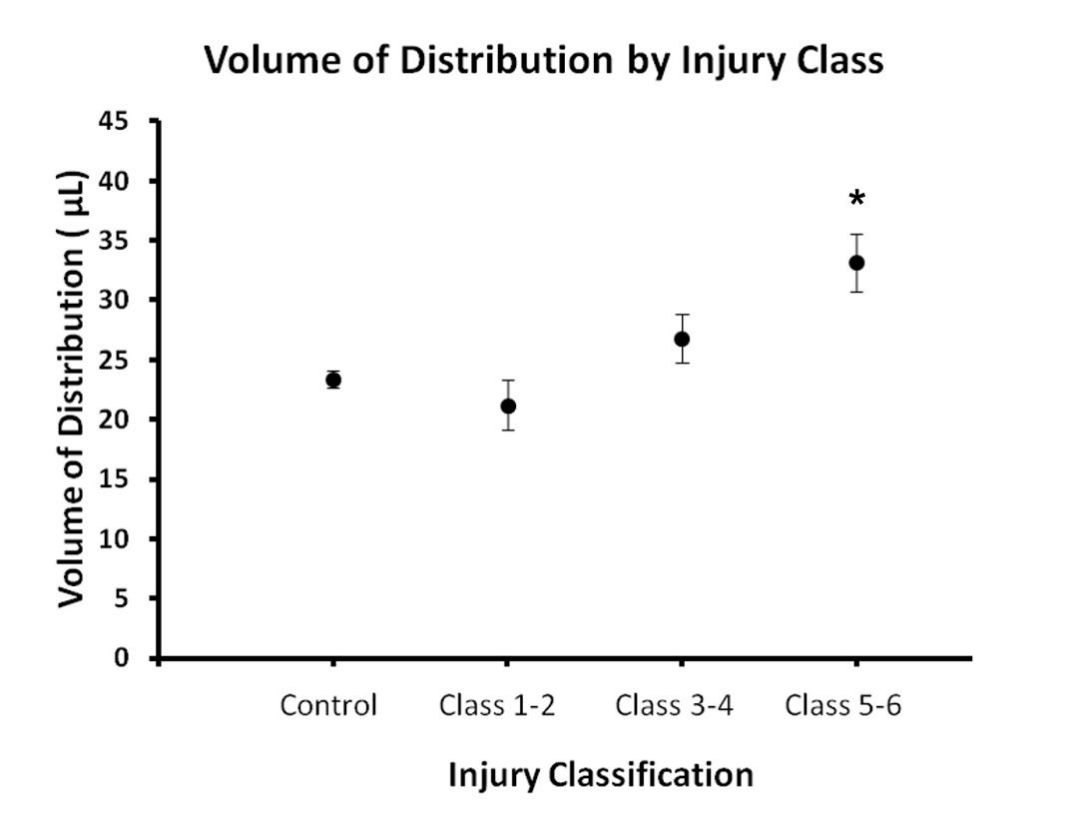
Increasing classifications of injury were correlated with volumes of distribution (tau = 0.51, p=0.006), which averaged 21.2 ± 3.6 µL for animals classified as Class 1-2, 26.8 ± 5.4 µL for animals classified as 3-4, and 33.2 ± 6.0 µL for animals classified 5-6. Distributions in animals Class 5 and above were significantly increased as compared to previously measured control animals (Class 0), which averaged 23.4 ± 1.8 µL (p=0.018, [6]).

Increasing classifications of injury were correlated with volumes of distribution of Gd-albumin in the brain (tau = 0.51, p=0.006), which averaged 21.2 ± 3.6 µL for animals classified as Class 1-2, 26.8 ± 5.4 µL for animals classified as 3-4, and 33.2 ± 6.0 µL for animals classified 5-6 (Figure 3). Distributions in animals with severe injury (Class 5 and above) were significantly increased as compared to previously measured control animals, which averaged 23.4 µL ± 1.8 µL [6] (p=0.018). This increase may be underestimated, as distribution volumes of control animals include an additional ~30 minutes of diffusion that occurred in the time delay between infusion and MR imaging. The contribution of diffusion was estimated to increase distribution volumes in control animals 2-3 voxels, or 0.250-0.350 mm [6]. No significant differences were found between animals presenting with ipsilateral versus contralateral parahippocampal injury.

Total hippocampal volumes in the brain pre-infusion were measured and compared to hippocampal volumes post-infusion. These volumes were not significantly different between injury classes (Class 1-2:18.4 ± 1.0, Class 3-4:18.6 ± 1.6, Class 5-6:19.2 ± 1.7), indicating that in this study, the larger infusion volumes seen in injured animals cannot be solely explained by differences in hippocampal size.

### Characteristics of Gd-albumin distribution

**Figure 4:**
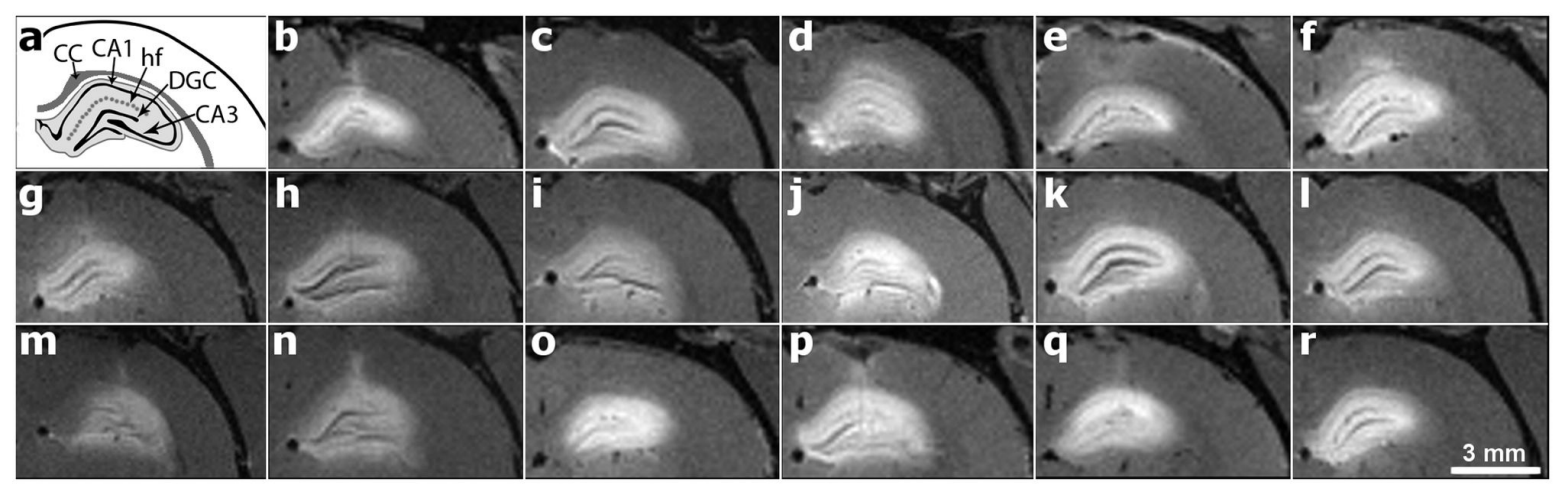
High-resolution T1-weighted images of Gd-albumin infusions into the dorsal hippocampus of 17 different rodent brains 24 hours post status epilepticus. **A**: Schematic of key structures in the dorsal hippocampus adapted from [44]. **B**-**R**: MR images of contrast agent distributions in the dorsal hippocampus. Hyperintense regions are voxels containing Gd-albumin. Distribution patterns contour along hippocampal circuitry with minimal backflow or exposure to extra-hippocampal regions. CC = Corpus callusom; CA1 = CA1 pyramidal cell layer; hf=hippocampal fissure; DGC = Dentate granule cell layer; CA3 = CA3 pyramidal cell layer.

While volumes of distribution correlated with injury classification (Figure 3), the pattern of infusate spread was consistent between animals exhibiting various levels of injury (Figure 4). All infusions showed clear demarcation within the hippocampus, with very minimal non-specific targeting due to backflow (flow of infusate back along the cannula track). Backflow was minimal in 9/17 (53%) animals, while 4 animals (24%) showed backflow within the corpus callosum overlying the hippocampal infusion site. Four animals exhibited no backflow at all. As previously described in control animals [6], spread of the contrast agent was seen in the dentate gyrus, CA3, CA2, CA1, and subiculum of the hippocampus. Additionally, both the dentate granule cell layer and pyramidal cell layers of CA3-CA1 and subiculum were clearly distinguishable from the surrounding hyperintense subfields, indicating poor contrast agent penetration. This was especially salient in the CA3 subregion in 6 animals (35%), where infusate coverage tapered at stratum pyramidale, not quite reaching stratum oriens (Figure 4B,F,G,K,L,R). In 4 animals (24%), infusate filled all layers of the CA3 subregion and also penetrated the fimbria (Figure 4I-J,M,P). As in controls, infusate did not extend to the contralateral hippocampus or any other subcortical structures in any animals; however, enhancement was observed as hyperintensity in T1 images within extraventricular regions surrounding the dorsal and ventral hippocampus. This is an additional route for non-specific targeting. Eleven animals (65%) exhibited leakage into the velum interpositum of the dorsal hippocampus, and 13 animals (76%) exhibited leakage into the midbrain cisterns of the ventral hippocampus, suggesting a portion of the contrast agent distribution was not accounted for within hippocampal distribution volumes. Eleven animals (65%) also exhibited leakage into the lateral ventricle ipsilateral to the infusion site.

### Histology

MR-visualized injury was corroborated and characterized using histological assessments of CNS injury (for summary, see Table 2). As control animals did not exhibit any noticeable staining of injury markers, positive staining was defined as a clear, visual presence of activated microglia, FJC, or CD68. This assessment served to confirm a neurological response to SE and provided a higher-resolution visualiztion of factors present in the extracellular space that may be contributing to CED distributions.

**Table 2:** Summary of the immunohistochemical results of hippocampal subregion injury per injury class.

		**Class 0**	**Class 1**	**Class 2**	**Class 3**	**Class 4**	**Class 5**	**Class 6**
**FJC**	CA3	-	-	+	+	+	+	+
	CA1	-	-	-	-	++	++	++
	Hilus	-	-	-	+	+	+	+
**CD-68**	CA3	-	-	+	+	+	+	+
	CA1	-	-	+	+	++	++	++
	Hilus	-	-	-	-	++	++	++
**GFAP**	CA3	-	-	+	+	+	+	+
	CA1	-	-	-	-	-	-	+
	Hilus	-	-	+	+	++	++

- Areas that do not exhibit injury marker

+ Areas exhibiting injury marker sparsely

++ Areas exhibiting injury marker densely

**Figure 5:**
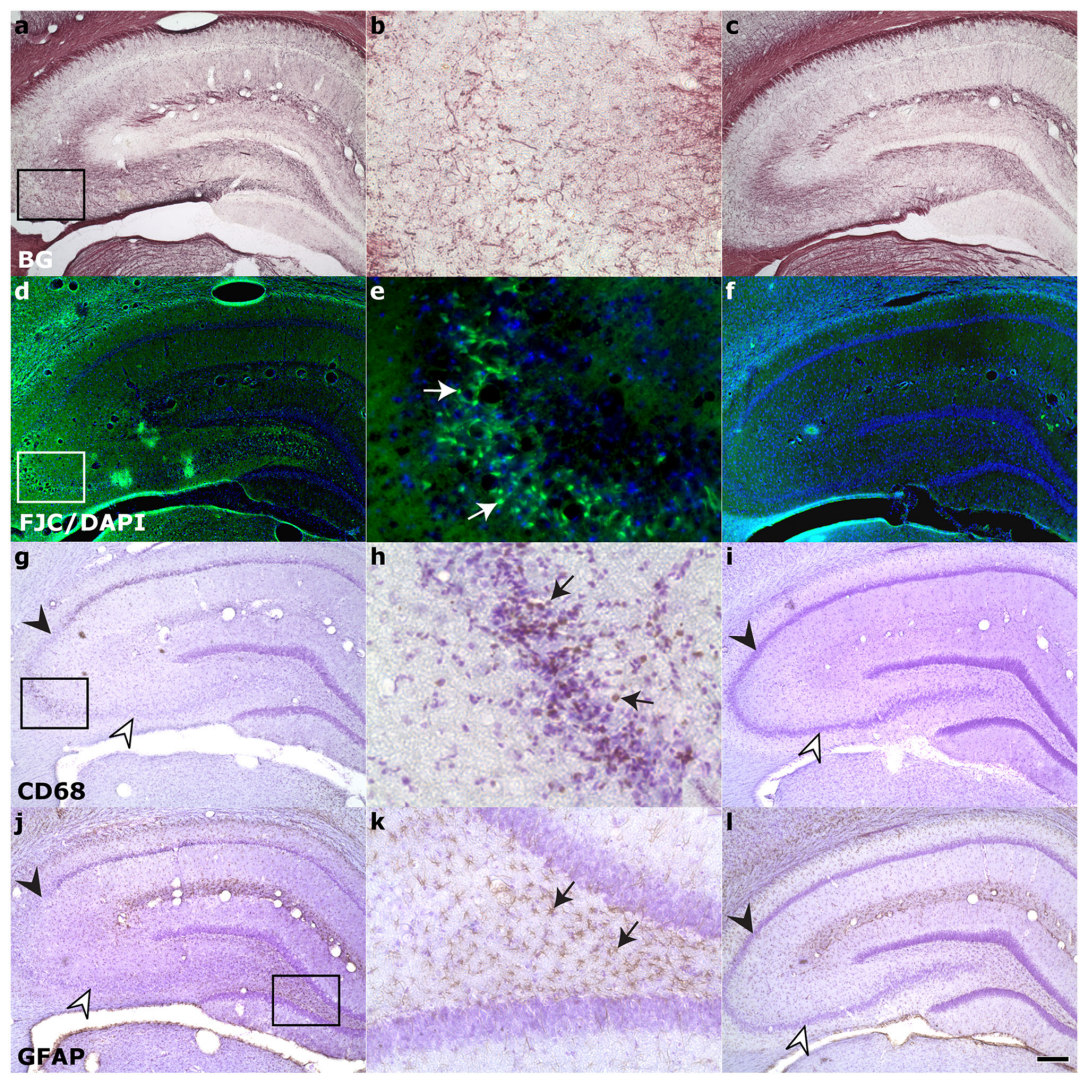
Characterization of hippocampal damage 24 hours post-status epilepticus. Representative stained sections of affected hippocampi (left column:low magnification, middle column:high magnification) are compared to unaffected hippocampi (right column) for **A**-**C**: myelin degradation, **D-F**: neuronal degeneration, **G-I**: macrophage activation, and **J**-**L**: astrocytosis. Higher magnification of boxed areas show ongoing neurodegeneration within CA3 (**E**, arrows) that corresponds with macrophage activation (**H**, arrows). Cell loss is corroborated through loss of cresyl violet staining in CA2 (closed arrowheads, compare **G,I** and **J,L**) and CA3 (open arrowheads, compare **G,I** and **J,L**). Astrocytosis was seen predominantly in the hilus (**K**, arrows), while myelin degradation was not appreciably different within the hippocampus (compare **A,C**). BG = Black-gold II; FJC/DAPI = Flouro-jade C with 4’,6-diamidino-2-phenylindole nuclear counterstain; GFAP = Glial fibrillary acidic protein; CD68 = Cluster of Differentiation 68; CV = Cresyl violet. Scale bar is 50µm for B,E,H,K; 200µm for all other images.

**Figure 6:**
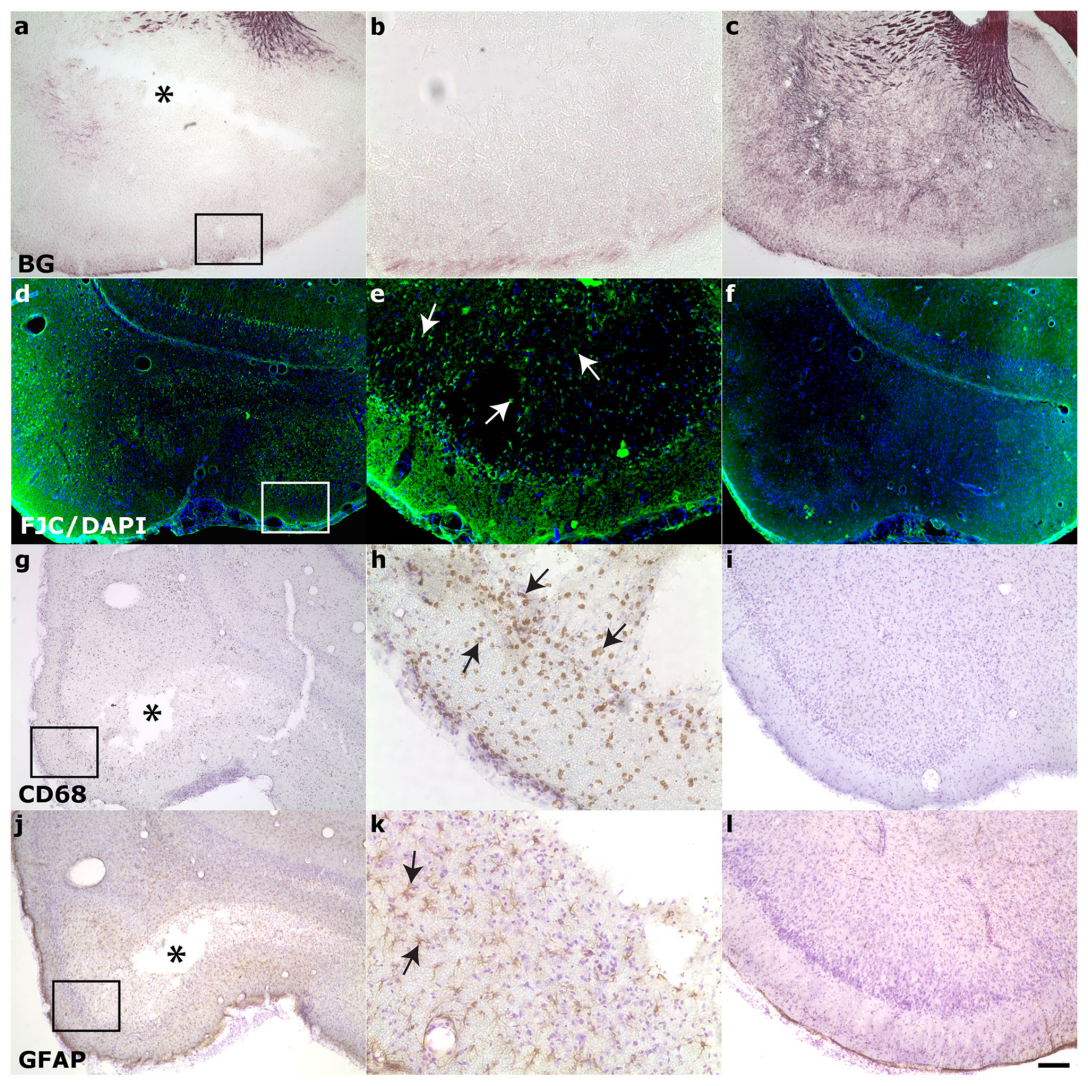
Characterization of parahippocampal damage 24 hours post status-epilepticus. Hyperintense regions observed in the parahippocampal region during in vivo T2-weighted imaging corresponded to **A**-**C**: myelin degradation, **D-F**: neuronal degeneration, **G-I**: macrophage activation, **J-L**: astrocytosis, and **A,G,J**: cavitation. Asterisks denote cavitation. Compare injured (left column:low magnification, middle column:high magnification) to uninjured (right column). BG = Black-gold II; FJC/DAPI = Flouro-jade C with 4',6-diamidino-2-phenylindole nuclear counterstain; GFAP = Glial fibrillary acidic protein; CD68 = Cluster of Differentiation 68; CV = Cresyl violet. Scale bar is 50µm for B,E,H,K; 200µm for all other images.

**Figure 7:**
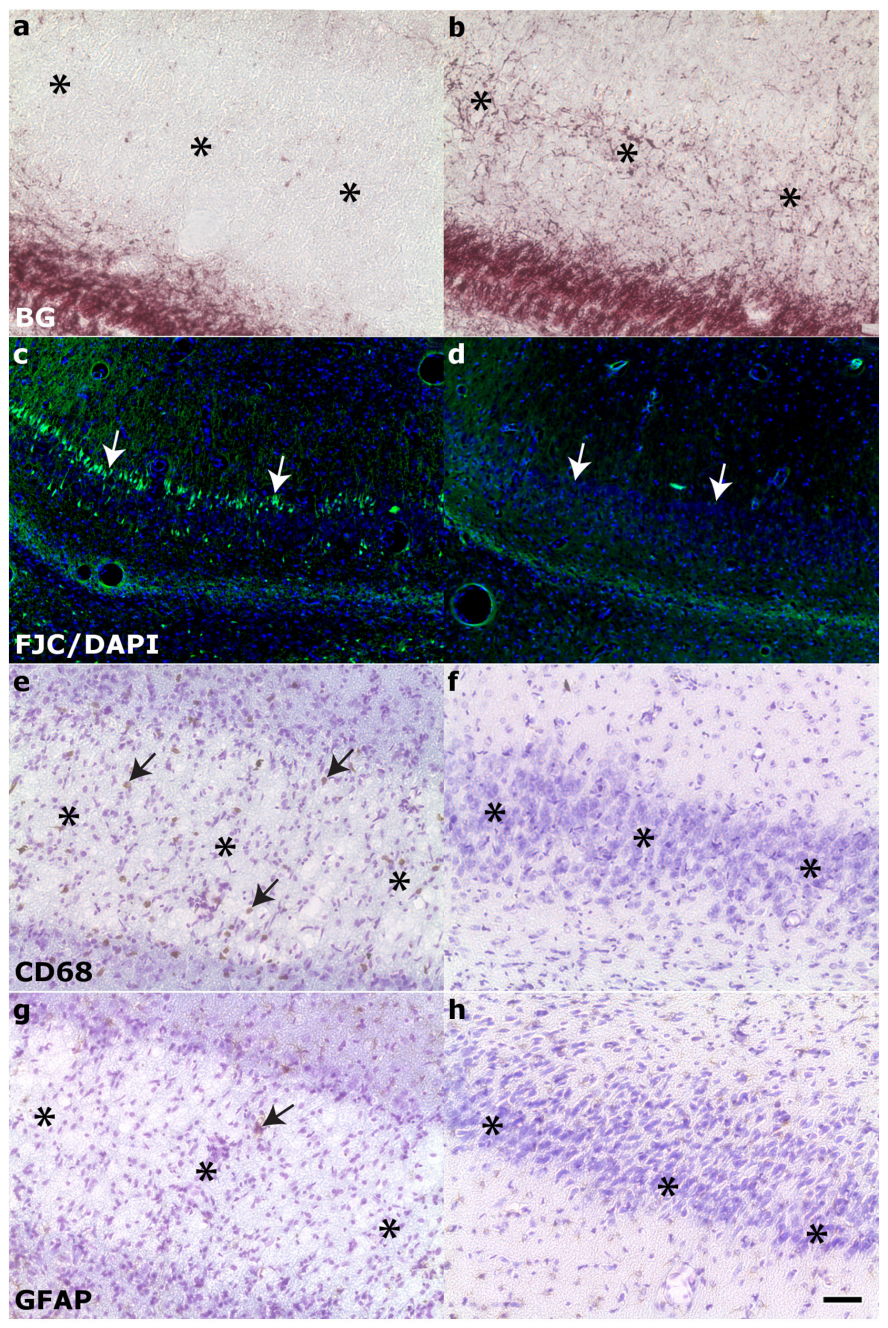
Damage to the ventral subiculum was seen in 6/17 rats 24 hours post status epilepticus. Affected (left column) is compared to unaffected hippocampi (right column). Injury in the ventral subiculum resulted in considerable **A-B**: myelin degradation, asterisks, and **C**-**D**: ongoing neuronal degeneration of the pyramidal cell layer, white arrows. **E**-**H**: Cresyl violet staining reveals significant cell layer degradation, asterisks. **E**,**F**: activated microglia, black arrows, were also seen in the ventral subiculum while **G**-**H**: total activated astrocytes were minimal, black arrows. BG = Black-gold II; FJC/DAPI = Flouro-jade C with 4',6-diamidino-2-phenylindole nuclear counterstain; GFAP = Glial fibrillary acidic protein, CD68 = Cluster of Differentiation 68; CV = Cresyl violet. Scale bar is 50µm.

**Figure 8:**
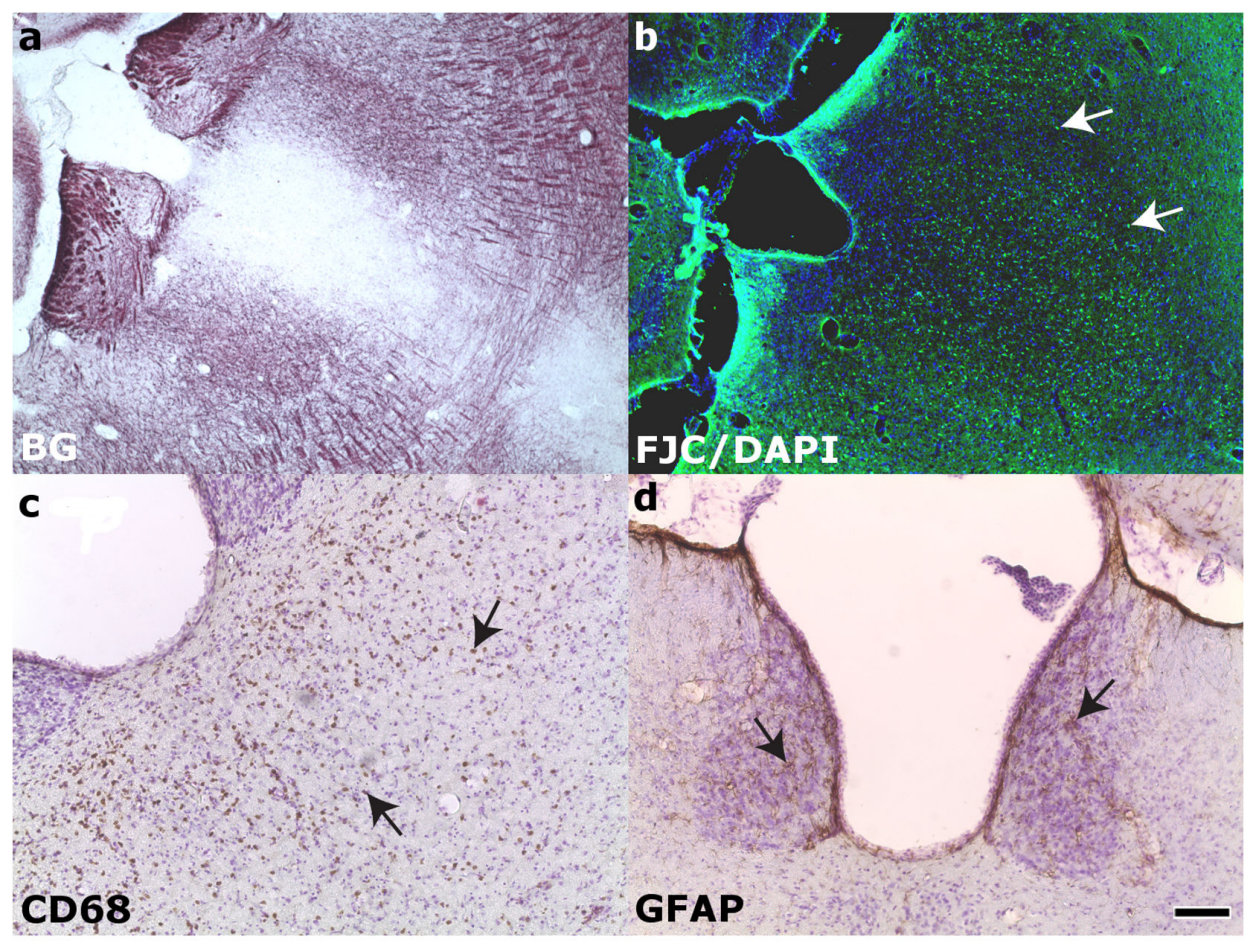
Thalamic injury 24 hours post status epilepticus. **A**: myelin staining did not show notable fiber degradation. **B**: ongoing neuronal degeneration and **C**: macrophage activation within middle thalamic nuclei. **D**: Activated astrocytes, arrows, were not seen in middle thalamic nuclei, but were present in middle habenular nuclei. BG = Black-gold II; FJC/DAPI = Flouro-jade C with  4',6-diamidino-2-phenylindole nuclear counterstain; GFAP = Glial fibrillary acidic protein, CD68 = Cluster of Differentiation 68; CV = Cresyl violet. Scale bar is 50µm.

Within the hippocampus proper (Figure 5), neurodegeneration was observed bilaterally via FJC staining in the CA3/CA2 subfield of all rats and in the hilus of rats with more severe injury (10/17 animals, Class 3-6). Positive FJC staining was not seen in the hippocampus of animals in injury Class 0-1 (compare Figure 5D,F). A minority (5/17) of animals in more severely injured classes (Class 4-6) also exhibited positive FJC staining in hippocampal subfield CA1 (Class 4-6). Phagocytic microgliosis was detected by expression of CD68 (Figure 5G-I, arrows) in the CA3 of 10/17 rats encompassing injury classes 2-6. Only a minority of animals exhibited activated microglia in the CA1 and hilus (5 rats in Classes 2-6 and 4/17 rats in Class 4-6, respectively). CD68-positive staining was not detected in animals of Class 0-1 (compare Figure 5G,I). GFAP staining was used to visualize the enlarged soma and processes typical of reactive astrocytes (Figure 5J-L, arrows). Hypertrophic astrocytes were seen in the hilus (Figure 5K, arrows) of the majority of rats (14/17 animals, Class 2-6) and in the CA3 of 5 rats (Class 2-6). Activated astrocytes were only seen in the CA1 of 1 rat in Class 6, the most severe injury class. All hyperintense regions identified in T2-weighted imaging were also immunostained for CNS injury markers. Hyperintensity observed in the parahippocampal region (Figure 6) during in vivo T2-weighted imaging corresponded to fluid-filled cavities (Figure 6, asterisks), myelin degradation (Figure 6A-C), neuronal degeneration (Figure 6D-F), macrophage activation (Figure 6G-I), and astrogliosis (Figure 6J-L). Injury in the ventral subiculum (Figure 7), which resulted in a classification of Class 5 or above, resulted in considerable degeneration of myelin (compare Figure 7A,B) and neurons, as measured by FJC staining (compare Figure 7C,D) and cell layer integrity in cresyl violet staining (compare Figure 7E,F and G,H). Thalamic injury (Figure 8) did not consist of notable myelin degradation (Figure 8A), but did encompass neuronal degeneration (Figure 8B, arrows) and microglia activation (Figure 8C, arrows). Activated astrocytes were seen in the medial habenular nucleus (Figure 8D, arrows).

No obvious difference in injury was observed in the hippocampi of rats that exhibited ipsilateral versus contralateral edema in the parahippocampal region. However, animals in higher classes of injury (4 and above) exhibited increased activation of FJC, CD68, and GFAP in the CA1 subfield, and increased activation of CD68 in the hilus (see Table 2). All animals exhibited injury near the electrode stimulation site at the ventral hippocampus. 

## Discussion

This study investigated the effect of acute injury on CED contrast-agent distribution profiles and volumes in the rat dorsal hippocampus following SE. CED distribution of Gd-albumin was visualized with high-resolution MR imaging, then tracer volume analysis was performed with segmentation. SE brain injury was rated based on edema visualized with T2-weighted MR images, and characterized with staining against neuronal degeneration, myelin degradation, astrocytosis, macrophage activation, and iron deposition. Volume of infusate distribution was increased in animals with more severe brain injury relative to mildly-brain injured animals and aged-matched controls. In normal hippocampi, CED performed under similar infusion conditions resulted in distribution volumes about 4.7 times greater than the volume infused [6], although these distribution volumes may be overestimated by an additional 30 minutes of diffusion. This spread corresponds to an estimated extracellular space volume percentage of 0.2 [50,51], which assumes an isotropic porous medium and no CSF leaks. The volumes of distribution in mildly injured animals in this study were similar to those measured in control animals (Class 0), with distribution volumes approximately 4.2 and 5.3 times greater than volumes infused in Class 1-2 and 3-4 animals, respectively. However, distribution volumes were significantly larger in animals that presented with a more severe CNS injury post-SE (Class 5-6), even with the presence of CSF leakage. For these animals, the distribution volume was 6.6 times larger than the volume of infusion. Distribution volumes may be increased by either decreases in extracellular space or increased extracellular flows that may occur with cytotoxic injuries. Edema, glial activation/swelling, and cellular degeneration were evident in the histological assessment, implying such injury may confer measurable effects on tissue distribution via changes in extracellular transport. 

### Injury and final infusate distribution volume

Following SE brain injury, a cascade of pathological events evolves over minutes, days, and weeks. Severity of CNS injury at 24 hours was classified in T2-weighted MR images and identified with several histological stains. Degeneration of neurons and myelin, in addition to activation of microglia and astrocytes was detected in the hippocampus, parahippocampal region, thalamus, and septal nuclei. Injury in these areas is consistent with other histological reports at acute time points ranging from 8 to 48 hours following administration of pilocarpine [52,53], kainate [28,54], angular bundle stimulation [55], and rostral forebrain stimulation [56]. 

The pathological states observed in this and other studies at acute time points include morphological changes, such as neuronal death, glial cell loss or proliferation, glial swelling, production of damaging metabolites, inflammation, edema, demyelination, and loss of ionic, pH, and amino acid homeostasis. They may also be accompanied by substantial changes in ECS ionic composition [57] and various changes in ECS diffusion parameters [51,57–60], which may result in reduction of the ECS in response to acute neuronal and glial swelling [58]. The presence of cellular debris and inflammatory markers paired with ECS changes increases the tortuosity, which is defined as the ratio of the diffusivity in free space to that in the brain. Changes in tortuosity reflect changes with edema and neuropil remodeling. This has been documented in several models of CNS injury, including cortical stab wounds [61], neural tissue grafts [62], and hypoxia [60], where the ECS volume fraction in rat cortex was specifically measured to decrease by 80% and was accompanied by an increase in tortuosity. Likewise, an acute insult resulting in cellular debris and/or the swelling of cells and fine glial processes would increase intercellular tortuosity, affect the size of the intercellular channels, and change extracellular transport and CED characteristics. This was observed in this study, where animals with higher classifications of injury displayed greater activation of macrophage presentation and neuronal degeneration, and they also exhibited larger volumes of distribution. We show the consequences of severe acute insults to the brain are increased CED distribution volumes, corroborating previous work demonstrating cytotoxic edema decreases the size of intercellular channels. An acute insult may also alter the integrity of the BBB [63], resulting in increased extracellular flow. Accordingly, the consequence of smaller intercellular channels and/or enhanced transport would be a larger volume of Gd-albumin CED distribution in rats that experienced a greater degree of injury. 

On the other hand, the microstructural injury present in the hippocampus at chronic time points (weeks-years) has different characteristics and perhaps opposite effects on infusate distribution. A longitudinal study using the SE animal model [17] showed that MR measurements in the hippocampi within acute periods (decreased average diffusivity (AD) and T2) were reversed at the onset of spontaneous seizures (increase in AD and T2). As opposed to the acute damage seen in this study, infusate spread in chronic TLE hippocampi may be much decreased, due to pooling of infusate in the increased ECS [64]. Further studies investigating the effect of other injury stages on distribution volumes are warranted, as they may have clinically relevant differences in how therapeutic agents need to be applied. Likewise, hippocampal injury in other disease states may also result in distinctive CED distributions. Future work involving models of varying pathologies could be used to explore the effect of such variability on infusate patterns in the hippocampus.

### Injury and pattern of infusate distribution

Largely underestimated, the biophysical properties of the local tissue architecture at the infusion site are a critical consideration for planning the coverage of therapeutic agents in both normal and injured areas of the brain. Even in normal rat brains, measured tortuosity in vivo was found to be higher in regions including a dense cell layer, such as stratum pyramidale, as compared to stratum radiatum, which contains mostly fibers [59]. Relatively isotropic, dense gray matter regions have been shown to affect CED distribution differently than directional white matter regions. Anisotropic regions such as axonal bundles often exhibit increased infusate transport along fiber directions[65] as compared to isotropic regions, while dispersion in laminar structures such as the hippocampus is harder to define due to variation of morphology over small spatial scales . In this study, we show that although infusate distribution volumes varied with brain injury, patterns of distribution within the dorsal hippocampus did not. Distribution patterns were predictable and consistent with those in control animals, spreading stereotypically throughout the hippocampus. Additionally, a majority of animals exhibited Gd-albumin leakage into the lateral ventricle, the velum interpositum, and the midbrain cisterns. Ventricles and perivascular spaces can act as mass sinks in which infusate may pool or be directed towards subarachnoid spaces [6,66], thereby reducing the volume of tissue exposed to infusate. The SE animal model showed similar leakage patterns as control animals, but other brain injuries known to influence these variables may need to consider their cannula targeting to avoid loss of infusate into ventricular and extra-ventricular spaces.

### Other factors affecting final infusate distribution

In this study, the severity of injury explains a portion of the variance seen in measured distribution volumes. There are also several other factors that may add to the remaining variability in the final distribution of infusate (for review, see 67). Infusion pump parameters, such as flow rate and duration of infusion [68,69],, and properties of the infusate itself, including size, shape, viscosity [70], solubility [71], binding characteristics, concentration, and rate of efflux from the brain will also influence interstitial transport. Aspects of the infusion methodology were purposely kept constant across animals in order to have a negligible effect on inter-subject variability. 

Backflow, which results in the spread of infusion into unintended regions, introduces variability in final distribution as well. This issue can be addressed with preventive cannula designs [72–74], but remains an important variable in drug delivery studies. White et al. [75] have shown that infusate distribution is dependent on catheter design and occurrence of tissue damage around the catheter. While the diameter used here was 53µm smaller than the thinnest outer diameter reported in White et al., an astroglial response was noted around the cannula track, with roughly half of infusions exhibiting very minimal backflow only along the cannula tract. About a quarter of infusions resulted in no backflow, and another quarter had backflow along the overlying corpus callosum. When compared to infusions with no backflow, the distribution volumes of infusions exhibiting backflow were not significantly affected (p=0.18).

Due to normal inter-animal structural variation, even small (≤1mm) differences in the exact cannula placement of each experiment may also factor into final distribution variability. Infusions in this study were targeted to the same stereotaxic coordinates in each animal; thus infusion site variability of the majority of animals was very minimal. However, two Class 4 animals with infusion sites too lateral in the hippocampus resulted in coverage that failed to spread into the medial CA1 subregion or the dorsal subiculum. This produced artificially low distribution volumes that fell within the average of Class 1-2 animals. Infusion site variability can be avoided with careful MR-guided cannula insertion, but emphasizes the effect of local tissue variability on infusate distribution.

### Future Goals for Clinical CED

 In light of the difficulties of CED delivery to the brain, significant work has been made toward applying this technique to human disorders. The hippocampus may be targeted using an MR-guided cannula or depth electrode. We have found that cannula placement within the stratum radiatum confers the best CED distributions, as the pyramidal and granule cell layers limit the spread of infusate to remain within the hippocampus. The corpus callosum presented a conduit to infusate spread, so white matter bundles should be avoided unless such spread is desired. Unfortunately, few studies have addressed neurological states of injury, excluding tumoral tissue. This study was an effort to measure CED infusions in edematous brain tissue, with the ultimate clinical goal of developing a MR-based therapy regimen that can be patient-specific. The results presented here show that edema created by hippocampal injury do in fact affect distributions of infusate, and are specific to the severity of injury. This suggests that the infusate concentration may also be altered, and thus the efficacy of an infused medication. In cases of severe cytotoxic edema, it may be advisable to adjust the treatment to a more concentrated therapeutic dose of smaller volume [76], as the injury response tends to increase the distribution volume. With further research, infusate parametrics can be established for optimal distribution patterns in other injury states as well.

## Conclusions

 This is the first study to observe CED delivery into an acutely injured hippocampus following limbic brain injury. This type of delivery bypasses systemic circulation and opens the possibility of therapy with a wide variety of potential therapeutic agents, since it does not require passage through the BBB or consideration of systemic safety. Several studies have described CED in normal hippocampi in different species, but understanding the influence of structural changes on extracellular transport in injured regions is critical for planning drug delivery studies for any neurological disorder. Historically, CNS injury has been associated with changes in the ECS, swelling of cellular elements, and overall increased tortuosity within the interstitial space. This was observed here as neuronal degeneration, myelin degradation, and macro- and microglia activation as an acute response to SE. Such changes have been previously determined to affect diffusion parameters, and in this study they correlated with CED distribution. These results will be incorporated in the planning of future studies tracking therapeutic agents and used for the improvement of computational models for CED infusions into injured brains.
