# Correction: Influence of Neuropathology on Convection-Enhanced Delivery in the Rat Hippocampus

**DOI:** 10.1371/annotation/803a76cf-2d5c-4ac0-9f48-c826cc09a4b3

**Published:** 2013-12-20

**Authors:** Svetlana Kantorovich, Garrett W. Astary, Michael A. King, Thomas H. Mareci, Malisa Sarntinoranont, Paul R. Carney

This paper was republished on December 19th due to a Table being erroneously incorporated into the text. 

